# A New Risk Score for Predicting Postoperative Mortality in Suspected Heart Failure Patients Undergoing Valvular Surgery

**DOI:** 10.31083/j.rcm2402038

**Published:** 2023-02-02

**Authors:** Hongyuan Lin, Jiamiao Gong, Kang An, Yongjian Wu, Zhe Zheng, Jianfeng Hou

**Affiliations:** ^1^Cardiac Surgery Centre, Fuwai Hospital, Chinese Academy of Medical Sciences and Peking Union Medical College, 100037 Beijing, China

**Keywords:** risk score, mortality, heart failure, valvular surgery

## Abstract

**Background:**

Heart failure (HF) is one of the most important indications of the severity of valvular heart disease (VHD). VHD with HF is frequently associated with a higher surgical risk. Our study sought to develop a risk score model to predict the postoperative mortality of suspected HF patients after valvular surgery.

**Methods:**

Between January 2016 and December 2018, all consecutive adult patients suspected of HF and undergoing valvular surgery in the Chinese Cardiac Surgery Registry (CCSR) database were included. Finally, 14,645 patients (55.39 ± 11.6 years, 43.5% female) were identified for analysis. As a training group for model derivation, we used patients who had surgery between January 2016 and May 2018 (11,292 in total). To validate the model, patients who underwent surgery between June 2018 and December 2018 (a total of 3353 patients) were included as a testing group. In training group, we constructed and validated a scoring system to predict postoperative mortality using multivariable logistic regression and bootstrapping method (1000 re-samples). We validated the scoring model in the testing group. Brier score and calibration curves using bootstrapping with 1000 re-samples were used to evaluate the calibration. The area under the receiver operating characteristic curve (AUROC) was used to evaluate the discrimination. The results were also compared to EuroSCORE II.

**Results:**

The final score ranged from 0 to 19 points and involved 9 predictors: age ≥60 years; New York Heart Association Class (NYHA) IV; left ventricular ejection fraction (LVEF) <35%; estimated glomerular filtration rate (eGFR) <50 mL/min/1.73 $m^{2}$; preoperative dialysis; Left main artery stenosis; non-elective surgery; cardiopulmonary bypass (CPB) time >200 minutes and perioperative transfusion. In training group, observed and predicted postoperative mortality rates increased from 0% to 45.5% and from 0.8% to 50.3%, respectively, as the score increased from 0 up to ≥10 points. The scoring model’s Brier scores in the training and testing groups were 0.0279 and 0.0318, respectively. The area under the curve (AUC) values of the scoring model in both the training and testing groups were 0.776, which was significantly higher than EuroSCORE II in both the training (AUC = 0.721, Delong test, *p *< 0.001) and testing (AUC = 0.669, Delong test, *p *< 0.001) groups.

**Conclusions:**

The new risk score is an effective and concise tool that could accurately predict postoperative mortality rates in suspected HF patients after valve surgery.

## 1. Background

Heart failure (HF) is a life-threatening condition and is associated with significant morbidity, poor functional capacity, and decreased quality of life [1]. In 2017, more than 64 million people worldwide were affected with HF [2], and the number is likely to rise. Savarese G *et al*. [3] reported in their survey that annual health care costs per HF patient amount up to €25,000 in the Western world, resulting in a substantial economic burden. The prevalence of valvular heart disease (VHD) among the elderly, as well as VHD-related HF, is rising as the population is ageing [4]. VHD is one of the most common types of cardiac surgery. According to a multicenter study conducted in China [5], the overall mortality rate for VHD surgery was around 2%. However, it is much higher in patients with HF, and can be greater than 3%. Nearly all risk prediction models for cardiac surgery include HF as an independent predictor. There is a growing demand for risk assessment for these surgical patients, however current risk scores do not provide a reliable estimate of the exact operative mortality in an individual HF patient [6, 7].

In order to better assess the risk of surgery for these patients, the aim of the present study is to establish a simplified scoring risk model based on the Chinese Cardiac Surgery Registry (CCSR) database to accurately predict the postoperative mortality of suspected HF patients undergoing VHD surgery.

## 2. Methods

### 2.1 Data Source

The CCSR is a multicenter registry, and consists of a council comprised of cardiac surgeons and researchers from the National Center of Cardiovascular Diseases which oversees the registry. This database contains information about cardiac surgery from 94 institutions. Each participating institution performed more than 100 cardiac surgeries each year and was requested to record cases using the same case report form (CRF). These sites are advanced cardiac centers and have many features that are common among large cardiac centers in China. According to the Chinese Society of Extracorporeal Circulation’s yearly surveys, we estimate that our database contains roughly 30% to 40% of all valvular procedures and represents surgical outcomes from large cardiac hospitals [8]. Every six months, two researchers investigated 5–10% of the reported cases at random for auditing. For cases in which there were missing data, the relevant participating units were required to resolve the problems in order to ensure the data’s integrity.

### 2.2 Patients

**Fig. 1.**
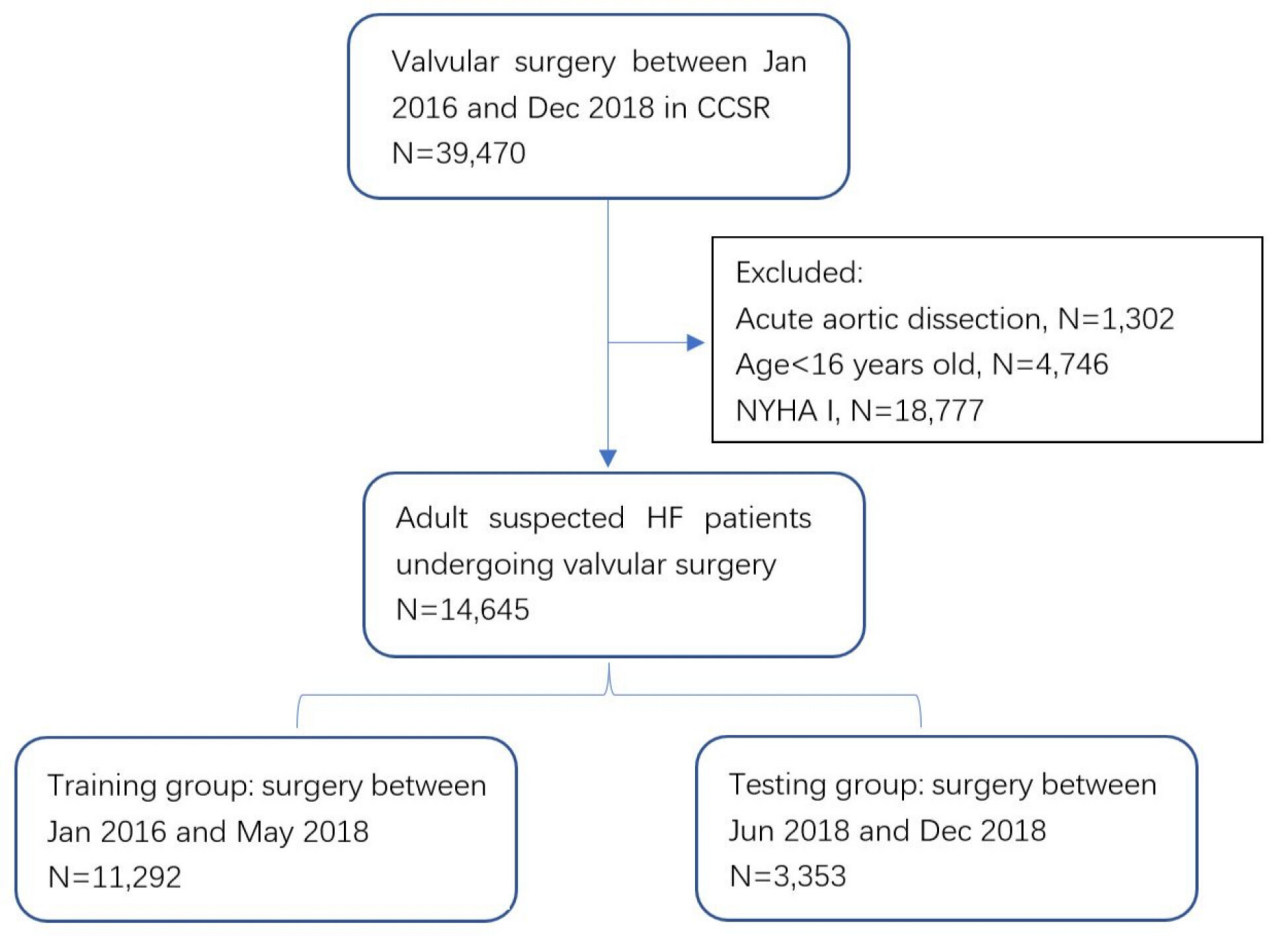
**Patient enrollment**. CCSR, Chinese Cardiac Surgery Registry; HF, heart failure; NYHA, New York Heart Association Class.

Between January 1, 2016, and December 31, 2018, we found 39,470 patients from the CCSR database who had undergone valvular surgery. We excluded 1302 individuals who had a primary diagnosis of acute aortic dissection, and whose hemodynamic characteristics were markedly different from those of VHD. We further removed 4746 patients under the age of 16 and 18,777 patients who had no HF related symptoms or signs and were classified as New York Heart Association Class (NYHA) I. Finally, we identified a total of 14,645 cases (NYHA II or higher) for analysis. Patients who had surgery between January 2016 and May 2018 (a total of 11,292) were allocated into the training group for model derivation. Patients who underwent surgery between June 2018 and December 2018 (a total of 3353) were included as a testing group to validate the model. The patient enrollment flow chart is shown in Fig. 1.

### 2.3 Definitions

We defined suspected HF patients as those who were classified as NYHA II or higher, due to valvular disease. According to the latest European Society of Cardiology (ESC) guidelines [1], suspected HF is defined as a clinical syndrome consisting of typical symptoms (e.g., breathlessness, ankle swelling, and fatigue) that may be accompanied by signs (e.g., elevated jugular venous pressure, pulmonary crackles, and peripheral oedema). It is due to structural or functional abnormalities of the heart that could result in elevated intracardiac pressures or inadequate cardiac output at rest or during exercise.

Postoperative mortality was defined as death occurring between the surgery and hospital discharge or within 30 days after surgery.

Definitions of other variables in Table 1 are shown in **Supplementary Table 1**.

**Table 1.** **Demographics and risk factors of training and testing groups, and according to vital status (30-day or in-hospital postoperative mortality) in training group**.

Variables	Training group (n = 11,292)	Testing group (n = 3353)	*p*-value	Training group		*p*-value of univariate analysis
				Alive (n = 10,958)	Death (n = 334)	
Patient related						
Age (years)	55.25 ± 11.6	55.87 ± 11.7	**0.007**	55.11 ± 11.6	59.91 ± 10.6	< **0.001**
Age ≥60 years	4254 (37.7)	1360 (40.6)	**0.003**	4060 (37.1)	194 (58.1)	< **0.001**
Female	4898 (43.4)	1471 (43.9)	0.619	4755 (43.4)	143 (42.8)	0.877
BMI (kg/$m^{2}$)	23.10 ± 3.35	23.09 ± 3.43	0.932	23.10 ± 3.34	23.10 ± 3.44	0.987
BSA ($m^{2}$)	1.65 (1.53–1.78)	1.64 (1.51–1.77)	0.081	1.65 (1.53–1.78)	1.63 (1.516–1.76)	0.381
Smoke	3583 (31.7)	999 (29.8)	**0.036**	3480 (31.8)	103 (30.8)	0.767
Diabetes mellitus	770 (6.8)	239 (7.1)	0.561	733 (6.7)	37 (11.1)	**0.002**
Hypertension	2881 (25.5)	826 (25.7)	0.838	2772 (25.3)	109 (32.6)	**0.003**
CKD	172 (1.5)	149 (4.4)	< **0.001**	155 (1.4)	17 (5.1)	< **0.001**
eGFR (mL/min/1.73 $m^{2}$)	88.49 (71.96–100.63)	89.08 (71.97–100.47)	0.962	88.70 (72.3–100.82)	80.06 (61.18–93.45)	< **0.001**
eGFR <50 mL/min/1.73 $m^{2}$	524 (4.8)	182 (5.4)	0.153	491 (4.5)	51 (15.3)	< **0.001**
Dialysis	31 (0.3)	16 (0.5)	0.099	22 (0.2)	9 (2.7)	< **0.001**
COPD	153 (1.4)	35 (1.0)	0.188	141 (1.3)	12 (3.6)	**0.001**
Extracardiac arteriopathy	213 (1.9)	29 (0.9)	< **0.001**	200 (1.8)	13 (3.9)	**0.011**
Previous stroke	527 (4.7)	133 (4.0)	0.095	497 (4.5)	30 (9.0)	< **0.001**
Heart related						
NYHA IV	825 (7.3)	215 (6.4)	0.083	763 (7.0)	62 (18.6)	< **0.001**
NYHA II or III	10,467 (92.7)	3138 (93.6)	0.083	10,195 (93.0)	272 (81.4)	< **0.001**
Chest pain	709 (6.3)	136 (4.1)	< **0.001**	659 (6.0)	50 (15.0)	< **0.001**
Arrhythmia	3576 (31.7)	960 (28.6)	**0.001**	3465 (31.6)	111 (33.2)	0.572
Critical status	95 (0.8)	43 (1.3)	**0.026**	83 (0.8)	12 (3.6)	< **0.001**
Previous myocardial infarction	360 (3.2)	78 (2.3)	**0.012**	336 (3.1)	24 (7.2)	< **0.001**
Previous cardiac surgery	564 (5.0)	149 (4.4)	0.209	528 (4.8)	436 (10.8)	< **0.001**
Previous valvular surgery	385 (3.4)	82 (2.4)	**0.006**	361 (3.3)	24 (7.2)	< **0.001**
LVEF (%)	54 (49–57)	54 (48–57)	**0.003**	54 (49–57)	51 (43–56)	< **0.001**
LVEF <35%	295 (2.6)	92 (2.7)	0.723	263 (2.4)	32 (9.6)	< **0.001**
Left main stenosis	305 (2.7)	89 (2.7)	0.932	277 (2.5)	28 (8.4)	< **0.001**
AS	2930 (25.9)	831 (24.8)	0.183	2853 (26.0)	77 (23.1)	0.246
Severe AI	2243 (19.9)	706 (21.1)	0.137	2182 (19.9)	61 (18.3)	0.5
MS	3956 (35.0)	1011 (30.2)	< **0.001**	3851 (35.1)	105 (31.4)	0.18
Severe MI	2392 (21.2)	771 (23.0)	**0.027**	2300 (21.0)	92 (27.5)	**0.005**
Severe TI	1126 (10.0)	384 (11.5)	**0.015**	1085 (9.9)	41 (12.3)	0.182
PS	46 (0.4)	9 (0.3)	0.32	44 (0.4)	2 (0.6)	0.903
Preoperative intravenous nitrate dependent	1157 (10.2)	230 (6.9)	< **0.001**	1098 (10.0)	59 (17.7)	< **0.001**
Preoperative intravenous catecholamine dependent	1054 (9.3)	184 (5.5)	< **0.001**	1012 (9.2)	42 (12.6)	**0.049**
RHD	5349 (47.4)	1603 (47.8)	0.67	5202 (47.5)	147 (44.0)	0.233
Active endocarditis	175 (1.5)	45 (1.3)	0.431	168 (1.5)	7 (2.1)	0.552
BNP (pg/mL)*	321.8 (265.2–288.7)	325.3 (268.9–290.2)	0.233	321.1 (264.6–279.8)	323.7 (267.3–289.4)	0.066
Operation related						
Non-elective surgery	144 (1.3)	59 (1.8)	**0.043**	119 (1.1)	25 (7.5)	< **0.001**
Aortic aneurysm operation	482 (4.3)	237 (7.1)	< **0.001**	455 (4.2)	27 (8.1)	**0.001**
CABG	1563 (13.8)	422 (12.6)	0.066	1450 (13.2)	113 (33.8)	< **0.001**
CPB time (minutes)	120 (92–159)	129 (99–167)	< **0.001**	120 (91–158)	167.5 (118.8–262.2)	< **0.001**
CPB time>200 minutes	857 (7.6)	343 (10.2)	< **0.001**	763 (7.0)	94 (28.1)	< **0.001**
AVR	6038 (53.5)	1796 (53.6)	0.941	5880 (53.7)	158 (47.3)	**0.025**
Aortic valvular repair	144 (1.3)	66 (2.0)	**0.004**	135 (1.2)	9 (2.7)	**0.036**
Mitral valvular surgery	7451 (66.0)	2118 (63.2)	**0.003**	7210 (65.8)	241 (72.2)	**0.018**
MVR	6246 (55.3)	1711 (51.0)	< **0.001**	6048 (55.2)	198 (59.3)	0.154
Aortic and mitral valvular surgery	2755 (24.4)	772 (23.0)	0.107	2669 (24.4)	86 (25.7)	0.604
Transfusion	6396 (56.6)	1881 (56.1)	0.591	6119 (55.8)	277 (82.9)	< **0.001**
EuroSCORE II	0.018 (0.012–0.028)	0.019 (0.012–0.029)	**0.043**	0.017 (0.011–0.027)	0.029 (0.020–0.052)	< **0.001**
Mortality	334 (3.0)	111 (3.3)	0.324	-	-	-

Values are presented as mean ± standard deviation, n (%), or median (interquartile range); bolded results are statistically significant (*p *< 0.05). BMI, body mass index; BSA, body surface area; eGFR, estimated glomerular filtration rate; CKD, chronic kidney disease; COPD, chronic obstructive pulmonary disease; NYHA, New York heart association; LVEF, left ventricular ejection fraction; AS, aortic valvular stenosis; AI, aortic valvular insufficiency; MS, mitral valvular stenosis; MI, mitral valvular insufficiency; TI, tricuspid insufficiency; PS, pulmonary valvular stenosis; RHD, rheumatic heart disease; BNP, Brain Natriuretic Peptide; CABG, coronary artery bypass grafting; CPB, cardiopulmonary bypass; AVR, aortic valve replacement; MVR, mitral valve replacement. *: BNP values were missing in about 40% of cases, the statistical results might not be reliable.

### 2.4 Statistical Analysis

We followed the TRIPOD (Transparent Reporting of a Multivariable Prediction Model for Individual Prognosis or Diagnosis) statement for reporting the derivation and testing of the prediction model [9]. Categorical variables were presented as frequencies (percentages %) and were compared with chi-squared tests. Continuous variables were presented as mean ± standard deviation (SD), and were compared with the *t* test or the Wilcoxon rank sum test as appropriate. A *p*-value < 0.05 was considered statistically significant. In the training group, all the possible risk factors were screened by univariate analyses and variables associated with a *p*-value < 0.05 level in univariate screening were entered into multivariate analysis, using a stepwise “both direction” procedure based on the Akaike information criterion (AIC), sequentially removing items until the lowest AIC was obtained. Continuous variables were dichotomized before entering into regression analyses by means of restricted cubic spline curves [10] (**Supplementary Figs. 1–4**) and/or accounting for clinically relevant thresholds. Regression coefficients of the final model were then used as weights to compute a simplified scoring system, by multiplying and rounding coefficients to their closest integer, following the approach from Cole to determine the optimal multiplier [11]. The area under the receiver operating characteristic curve (AUROC) was used to evaluate model discrimination, and calibration curves were plotted to assess the concordance between observed and anticipated probabilities. 1000 bootstrap re-samples were used for validation. In addition, in terms of Brier score for calibration and AUROC for discriminating, the risk model was compared to EuroSCORE II. The Delong test was used to compare AUC values (with 95 percent confidence interval, 95% CI). R software version 4.0.3 (R Foundation for Statistical Computing, Vienna, Austria) was used for statistical analysis. GraphPad Prism version 8.0 (GraphPad Software, San Diego, CA, USA) was used to draw figures.

## 3. Results

### 3.1 Study Population

Fig. 1 illustrates the patient enrollment flow chart. Table 1 compares the demographics and other pre- or intraoperative risk variables of the training group (n = 11,292) with the testing group (n = 3353). **Supplementary Table 1** contains the definitions of the variables in Table 1. The mean age in the training group was 55.25 and was 55.87 in the testing group (*p* = 0.007). The training group had 4898 (43.4%) female patients, while the testing group had 1471 (43.9%) female patients (*p* = 0.619). In the whole cohort, a total of 7834 patients (53.5%) and 7957 patients (54.3%) received AVR and MVR procedures, respectively. Furthermore, only 1612 patients (11%) had an MV repair operation. Patients with concomitant moderate to severe tricuspid insufficiency were simultaneously performed with tricuspid repair operation. In the training group, the median EuroSCORE II value was 0.018, while in the testing group, it was 0.019 (*p* = 0.043). The postoperative mortality rate was 3% in the training group and 3.3% in the testing group (*p* = 0.324).

### 3.2 Univariate and Multivariate Analyses

Factors associated with postoperative mortality after univariate screening are presented in Table 1. To construct a simplified scoring system, continuous variables were dichotomized before analyses and were defined as follows: age ≥60 years, eGFR <50 mL/min/1.73 $m^{2}$, CPB time >200 minutes and LVEF <35%.

Table 2 shows results of the multivariate analysis. The independent variables selected to construct the final model were: age ≥60 years; NYHA IV; left ventricular ejection fraction (LVEF) <35%; estimated glomerular filtration rate (eGFR) <50 mL/min/1.73 $m^{2}$; preoperative dialysis; left main artery stenosis; non-elective surgery; cardiopulmonary bypass (CPB) time >200 minutes and perioperative transfusion. In the final simplified scoring model, points attributed to each predictor according to its odds ratio are likewise presented in Table 2.

**Table 2.** **Risk factors for 30-day or in-hospital all-cause death: final model from multivariate analysis and scoring system**.

Risk factors	Odds ratio	95% CI	Regression coefficient	Final scoring
Age ≥60 years	2.07	1.65–2.63	0.73	2
NYHA IV	1.75	1.49–2.80	0.56	1
LVEF <35%	2.83	2.04–4.75	1.04	2
eGFR <50 mL/min/1.73 $m^{2}$	2.18	1.60–3.27	0.78	2
Dialysis	4.30	1.50–9.89	1.46	4
Left main artery stenosis	1.79	1.26–3.02	0.59	1
Non-elective surgery	3.57	2.32–6.56	1.27	3
CPB time >200 minutes	3.47	2.78–4.78	1.25	3
Transfusion	1.99	1.14–2.98	0.69	1

CI, confidence interval; NYHA, New York heart association; LVEF, left ventricular ejection fraction; eGFR, estimated glomerular filtration rate; CPB, cardiopulmonary bypass.

### 3.3 Model Validation

**Fig. 2.**
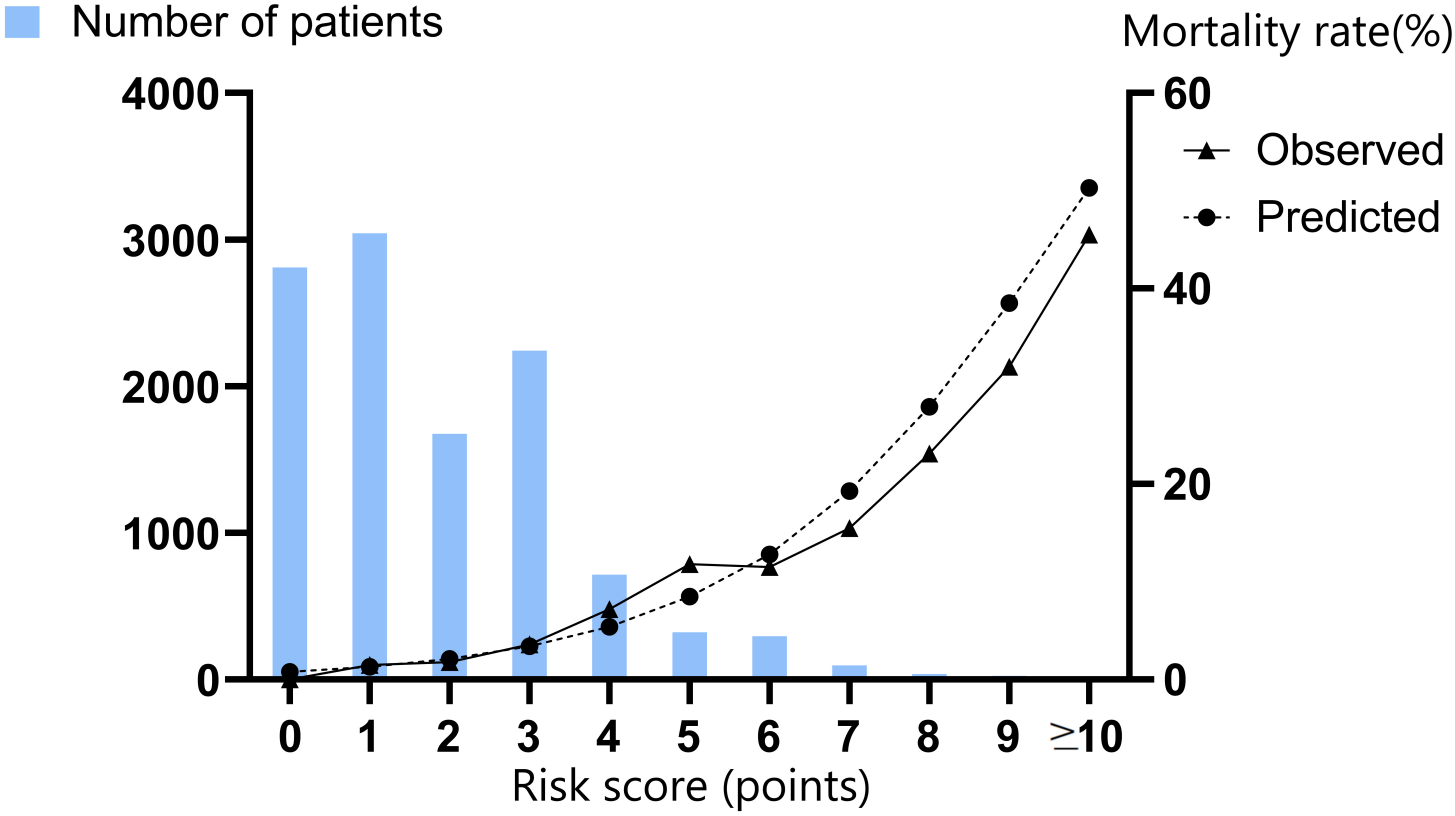
**Predicted vs. observed mortality rates and numbers of patients according to the risk score value (in training group)**.

Observed and predicted in-hospital mortality rates according to the score from the simplified scoring model ranged from 0% to 45.5% and from 0.8% to 50.3%, respectively, for a score of 0 to 10 or more, with exponential increasing mortality rates as the score increased (Table 3 and Fig. 2).

**Table 3.** **Predicted vs. observed mortality rates according to the score value (in training group)**.

Score	Number of patients	Predicted mortality rate (%)	Observed mortality rate (%)
0	2811	0.8	0
1	3043	1.3	1.5
2	1677	2.1	1.8
3	2244	3.4	3.6
4	715	5.4	7.2
5	322	8.5	11.8
6	297	12.8	11.5
7	97	19.3	15.5
8	39	27.9	23.1
9	25	38.5	32
≥10	22	50.3	45.5

**Fig. 3.**
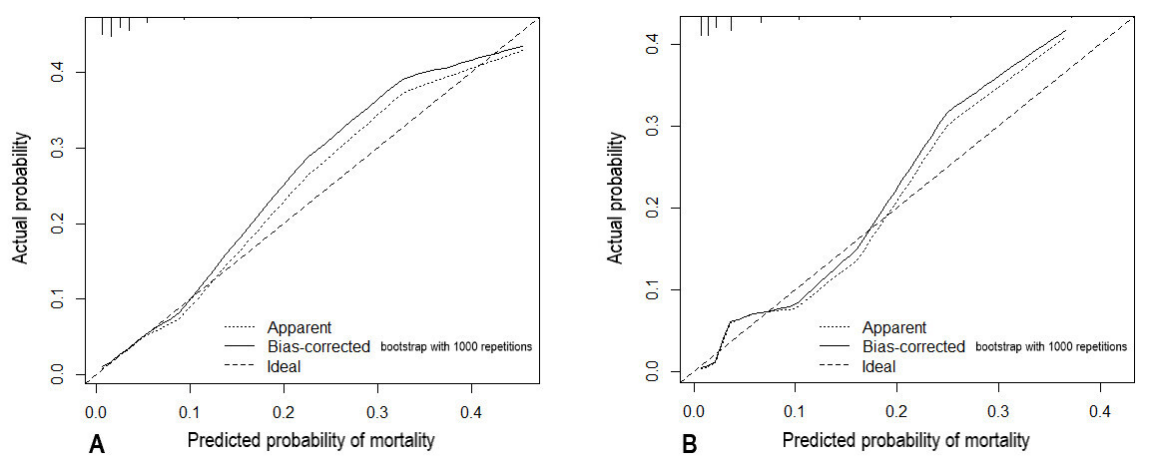
**Calibration curves of the risk score model: predicted vs. actually observed probability of mortality (A: training group; B: testing group)**.

The calibration of the risk score model was good, as shown in Fig. 3, exhibiting satisfied agreement between observed and predicted probability of mortality for probabilities up to 40%, with a slight underestimation of this model for probabilities ranged from 20% to 40%.

Fig. 3 shows the calibration plots of the simplified scoring model, and it can be seen that the calibration of the model is satisfactory in both training and testing groups.

**Fig. 4.**
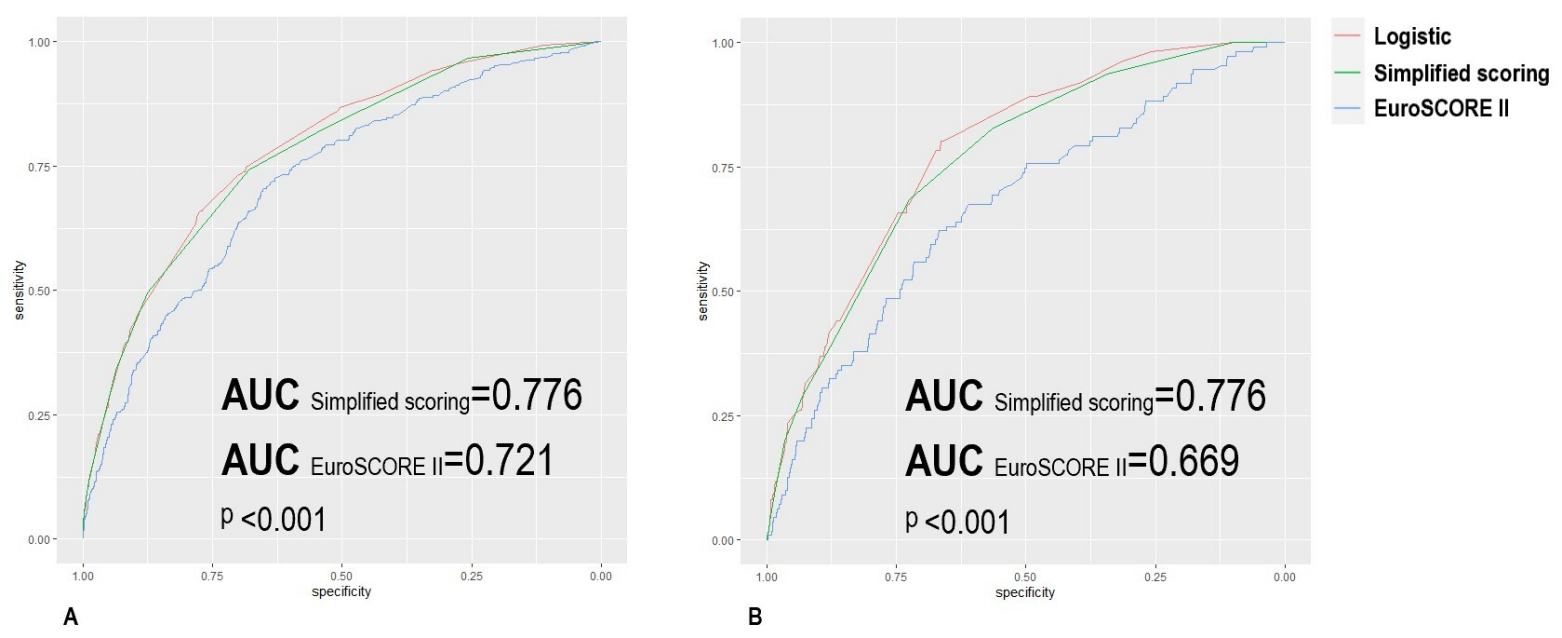
**Receiver operating characteristic curves from final logistic model: simplified risk score model and EuroSCORE II (A: training group; B: testing group)**.

In the training set, the AUC of our simplified scoring model was 0.776, which was statistically higher than EuroSCORE II with an AUC of 0.721 (Delong test, *p *< 0.001). Meanwhile, the Brier score of our model was 0.0274, lower than EuroSCORE II (0.0279). The comparison of ROC curves is shown in Fig. 4A.

In the testing group, the AUC of our simplified scoring model was 0.776, which was remarkably higher than EuroSCORE II with an AUC of 0.669 (Delong test, *p *< 0.001). Meanwhile, the Brier score of our model was 0.0308, also lower than EuroSCORE II (0.0318). The comparison of ROC curves is shown in Fig. 4B.

Tables 4,5 show the AUCs and Brier scores of two models.

**Table 4.** **Area under curve (AUC) values of final multivariate logistic model, simplified risk score model and EuroSCORE II: AUCs (95% CI)**.

	Logistic	Simplified scoring	EuroSCORE II	*p* value of Delong test (simplified scoring vs. EuroSCORE II)
Training group	0.784 (0.76–0.809)	0.776 (0.75–0.8)	0.721 (0.638–0.75)	<0.001
Testing group	0.786 (0.747–0.824)	0.776 (0.736–0.816)	0.669 (0.617–0.722)	<0.001

**Table 5.** **Brier scores of final multivariate logistic model, simplified risk score model and EuroSCORE II**.

	Logistic	Simplified scoring	EuroSCORE II
Training group	0.0271	0.0274	0.0279
Testing group	0.031	0.0308	0.0318

**Fig. 5.**
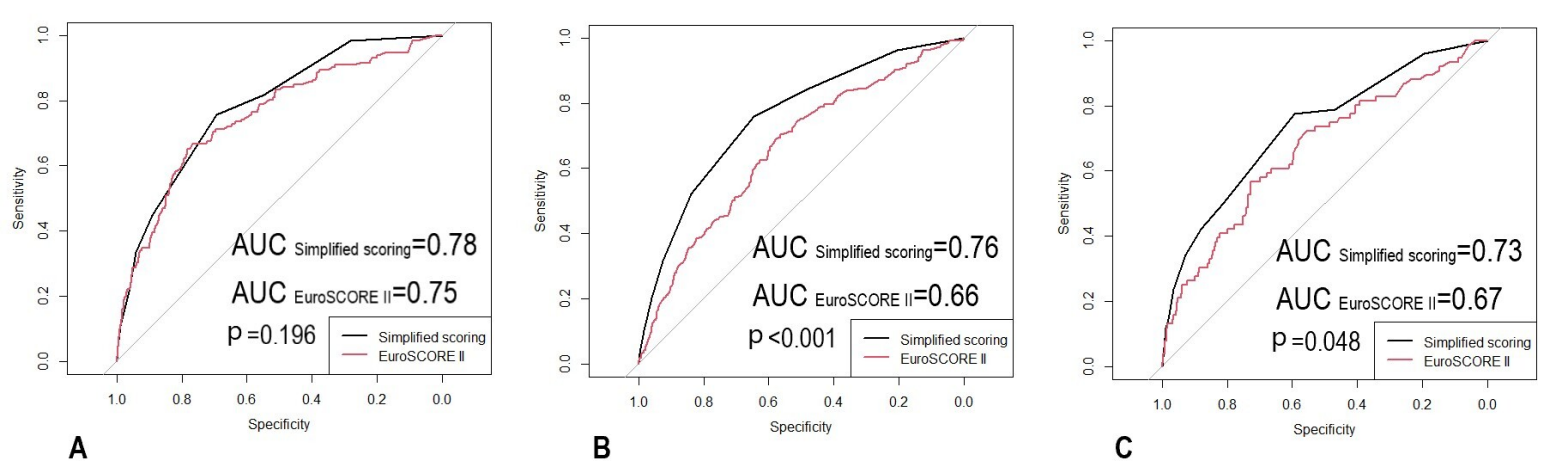
**Receiver operating characteristic curves from our simplified scoring and EuroSCORE II in subgroups (A: NYHA II, n = 5594; B: NYHA III, n = 8011; C: NYHA IV, n = 1040)**.

Interestingly, we found the difference of performance between our simplified scoring model and EuroSCORE II might increase according to the degree of HF presented by the patients. Fig. 5 shows comparisons of ROC curves between two scores validated in subgroups of different NYHA classifications.

## 4. Discussion

This risk score is an effective and simple tool for mortality prediction after valvular surgery in patients with HF. Unlike the EuroSCORE II, which has 18 predictors, this model includes only 9 predictors which are easily accessible in clinical practice. The model is convenient for clinical use and could be a reliable bedside tool.

HF is a major health-care issue that is related to high resource usage and health-care costs [3]. HF is also the leading cause of hospitalization in people over the age of 65 [12]. VHD is amongst the most common primary causes of HF, and many VHD patients require surgery. HF has long been a focus of clinical perioperative evaluation as an independent risk factor for cardiac surgery. The definitive diagnosis of HF, on the other hand, is challenging, especially for HF with preserved LVEF, which necessitates a combination of clinical symptoms and signs, as well as a variety of objective laboratory and ultrasound indicators. Many patients cannot receive a precise diagnosis of HF prior to surgery due to the wide discrepancies in preoperative examination of VHD patients among different cardiac institutes in China. The target population of this study was therefore identified as suspected HF, which could be quickly diagnosed based on symptoms, signs, and valvular abnormalities, thereby enhancing the clinical application of this model. Previous prediction models may no longer be able to reliably estimate current surgical risk due to improvements in surgical techniques and perioperative treatment. Prediction models are time-sensitive: an excellent prediction model must be continuously updated. For instance, consider EuroSCORE II was released in 2012, and has nearly fully replaced EuroSCORE I, which was first published in 1999. Therefore, in our study, we developed a prediction model based on the most recent clinical data that could objectively reflect current VHD features and surgical outcomes.

Furthermore, the prediction model is region-specific, because people in different regions of the world have distinct disease features [13], and there is regional variation in therapeutic concepts and techniques [6, 14]. Currently, the most of widely used clinical prediction models (such as EuroSCORE II and the society of thoracic surgeons (STS) score) were based on western populations. These western models may not be ideal for Asia or the Chinese population. The EuroSCORE II has underperformed in the Chinese suspected HF population, according to our findings. Our subgroup analysis (Fig. 5) indicates that in terms of discrimination, our model was significantly better than EuroSCORE II among NYHA III or IV patients. As a result, developing a prediction model for Chinese suspected HF patients who require VHD surgery is important in clinical practice. Wessler *et al*. [6] published a study showing that many VHD prediction models performed poorly in validation. They suggested that one probable explanation is a lack of sample size or a poor representative of the sample population for model derivation. Fortunately, one of the advantages of our study is that the sample population is well-representative using the CCSR data. The CCSR is the largest Chinese multicenter cardiac surgery database, analogous to the STS in North America, and includes almost all high-quality cardiac hospitals in China. As a result, this risk model’s validation performance was satisfactory, and considerably better than EuroSCORE II. However, pending testing and practice in real world, this score’s clinical significance will have to be determined for other populations.

Preoperative renal function indicators (eGFR and prior dialysis) and cardiac function indicators (NYHA IV and LVEF <35%) account for the large proportion of predictors in this model. The renal function indicators have the highest weights, implying that renal function has a significant impact on the prognosis of HF patients. Metra M *et al*. [15] suggested that worse renal function might result in poorer clinical outcomes in HF patients. And a meta-analysis [16] showed inadequate renal function was found in 23% of the HF patients, and was associated with a two-fold increased risk of all-cause death, with greater magnitude of the association whether LVEF was higher. In those who suffer from HF, preoperative CKD is a predictor of poor outcomes in all cardiac surgical patients and nearly all cardiac surgery prediction models include preoperative renal function as a predictor. In contrast to coronary artery bypass grafting (CABG) surgery, there are many different valvular surgical techniques. The surgical method, however, is not an independent risk factor for postoperative mortality, as shown in this study. It indicates that, as valvular surgical techniques have advanced in recent years, the effects of disparities between surgical methods on prognosis are decreasing, highlighting the importance of the patient’s underlying medical conditions in determining prognosis.

The predictive probability of many existing VHD surgical risk models is not good [7, 17]. One of the reasons might be the diversity of VHD surgical methods and the relatively small sample sizes for model derivation [18]. Some investigations had proposed that, in addition to traditional factors, risk models should include more predictors to increase their effectiveness [7]. Given the vast number of intraoperative uncertainties in VHD surgery, we added certain essential intraoperative predictors to the model, in addition to some fundamental preoperative variables, to improve the model’s prediction capability. CPB time, for example, was chosen as a predictor in this study because CPB time provides a thorough reflection of surgical complexity and surgeon proficiency. The longer the CPB time, the more complex the surgery and/or the less skilled the surgeon.

Our risk score model’s primary goal is to offer patients and health care practitioners more accurate information about the risk of VHD surgery and to aid in decision-making. This simplified score model is simple to use, as it is based on nine predictors that are routinely accessed in VHD patients. When considering VHD surgery, it aids in stratifying the risk of mortality.

## 5. Limitations

There is still a gap in nationwide representativeness between CCSR and STS. CCSR includes only data from high-quality cardiac centers in China, hospitals with lower operation volumes are not included. A definitive diagnosis of HF requires objective laboratory and ultrasound indicators. Unfortunately, there are many missing data of these indicators in the current database. Although this model can be used for preoperative evaluation, it is not a complete preoperative evaluation model due to the inclusion of intraoperative predictors. This model also needs external validation in real world practice to evaluate its clinical applicability. In addition, although all patients had HF at admission, after preoperative medical treatment, some of them had improved cardiac function by the time of surgery, and this updated information might not be collected in time. As a result, the data in CCSR might not actually reflect the latest status of every patient before surgery, and is one of the major limitations of this study.

## 6. Conclusions

The new risk score is an effective and concise tool that could accurately predict postoperative mortality rates in suspected HF patients after valve surgery.

## Data Availability

The CCSR registry is not publicly available, but the datasets used and/or analysed during the current study are available from the corresponding author on reasonable request. The permission to access and use the raw data was granted by Prof. Shengshou Hu and from the Fuwai hospital.

## Supplementary Material

Supplementary material associated with this article can be found, in the online version, at https://doi.org/10.31083/j.rcm2402038.
