# Supplementary material for: A New Risk Score for Predicting Postoperative Mortality in Suspected Heart Failure Patients Undergoing Valvular Surgery
Source: Rev Cardiovasc Med. 2023 Feb 2;24(2):38. doi: 10.31083/j.rcm2402038 (PMC11273104; doi:10.31083/j.rcm2402038)

Supplementary table 1. Definitions of variables

| Variables | Definitions |
| --- | --- |
| Age (years) | - |
| Female | - |
| BMI (kg/ m^2^) | Body mass index |
| BSA (m^2^) | Body surface area |
| Smoke | Prior history of smoking, regardless of whether the patients quit smoking |
| Diabetes mellitus | Documented past history or fulfilled the criteria of WHO 1999 |
| Hypertension | Documented past history or SBP > 140 mmHg and/or DBP > 90 mmHg |
| CKD | Documented past history or fulfilled the criteria of KDIGO 2012 |
| eGFR (ml/min/1.73 m^2^) | Estimated by the Modification of Diet in Renal Disease (MDRD) equation |
| Dialysis | Documented past history |
| COPD | Long-term use of bronchodilators or steroids for lung disease |
| Extracardiac arteriopathy | Any one or more of the following: claudication, carotid occlusion or>50% stenosis, previous or planned intervention on the abdominal aorta, and limb arteries or carotids |
| Previous stroke | Documented past history of coma ≥24 h or central nervous system dysfunction ≥72 h |
| NYHA IV | NYHA classification |
| Chest pain | Documented past history |
| Arrhythmia | Atrial fibrillation, flutter or atrioventricular block within 2 weeks before operation |
| Critical status | Any one or more of the following occurring preoperatively: ventricular tachycardia or fibrillation or aborted sudden death; cardiac massage; ventilation before arrival in the anaesthetic room; intra-aortic balloon counterpulsation or ventricular-assist device before arrival in the anaesthetic room; acute renal failure (anuria or oliguria <10 ml/h) |
| Previous myocardial infarction | Documented past history |
| Previous cardiac surgery | One or more previous major cardiac operation involving opening the pericardium |
| Previous valvular surgery | One or more previous major cardiac valvular operation |
| LVEF (%) | Assessed by echocardiography (measured before surgery) |
| Left main stenosis | Left main coronary artery stenosis>50% |
| AS | Assessed by echocardiography (measured before surgery) |
| Severe AI | Assessed by echocardiography (measured before surgery) |
| MS | Assessed by echocardiography (measured before surgery) |
| Severe MI | Assessed by echocardiography (measured before surgery) |
| Severe TI | Assessed by echocardiography (measured before surgery) |
| Preoperative intravenous nitrate dependent | Need intravenous nitrate to keep hemodynamic stability before surgery |
| Preoperative intravenous catecholamine dependent | Need intravenous catecholamine to keep hemodynamic stability before surgery |
| RHD | Documented past history |
| Active endocarditis | Patients still on antibiotic treatment for endocarditis at the time of surgery |
| Non-elective surgery | Not routine admission for operation |
| Aortic aneurysm operation | Combined with aortic aneurysm (or dissecting aneurysm) operation |
| CABG | Combined with CABG operation |
| CPB time (minutes) | Cardiopulmonary bypass time |
| AVR | Aortic valve replacement |
| Aortic valvular repair | - |
| Mitral valvular surgery | - |
| MVR | Mitral valve replacement |
| Aortic and mitral valvular surgery | - |
| Transfusion | Transfusion before or during surgery |

BMI: body mass index; BSA: body surface area; WHO: world health organization; SBP: systolic blood pressure; DBP: diastolic blood pressure; CKD: chronic kidney disease; KDIGO: Kidney Disease Improving Global Outcomes; eGFR: estimated glomerular filtration rate; COPD: chronic obstructive pulmonary disease; NYHA: New York heart association; LVEF: left ventricular ejection fraction; AS: aortic valvular stenosis; AI: aortic valvular insufficiency; MS: mitral valvular stenosis; MI: mitral valvular insufficiency; TI: tricuspid insufficiency; PS: pulmonary valvular stenosis; RHD: rheumatic heart disease; CABG: coronary artery bypass grafting. CPB: cardiopulmonary bypass; AVR: aortic valve replacement; MVR: mitral valve replacement


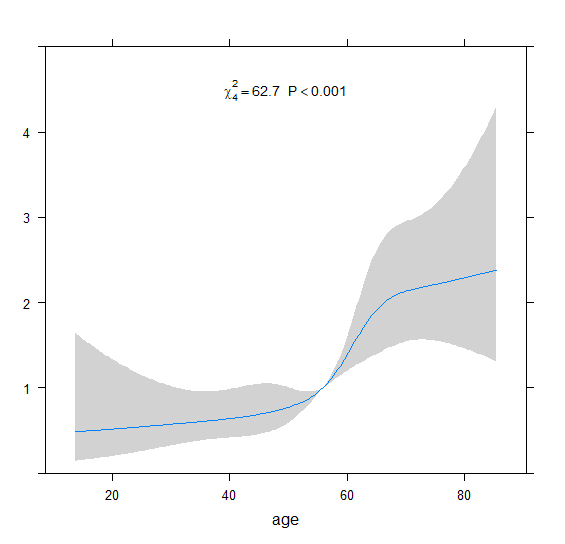
Supplementary fig. 1. Restricted cubic spline curve: age (years)

Supplementary fig. 2. Restricted cubic spline curve: eGFR (ml/min/1.73 m^2^)


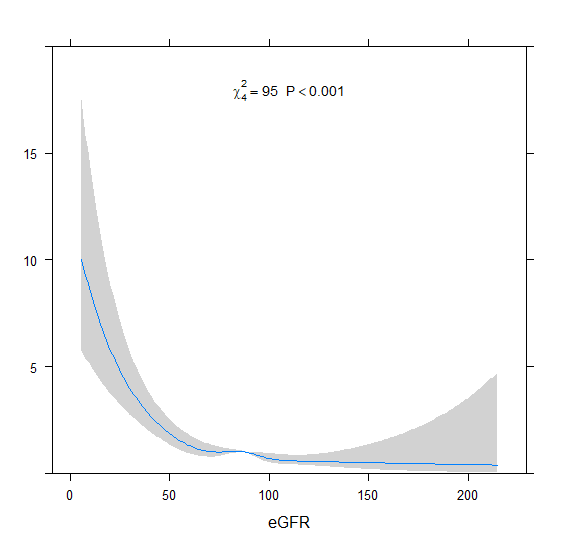


Supplementary fig. 3. Restricted cubic spline curve: LVEF (%)


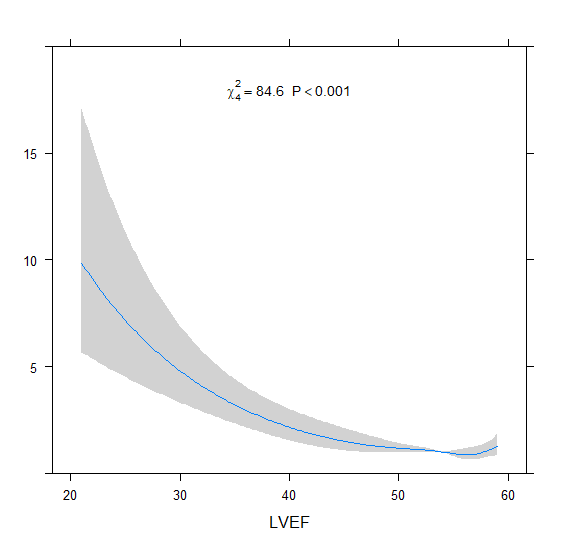


Supplementary fig. 4. Restricted cubic spline curve: CPB time (minutes)


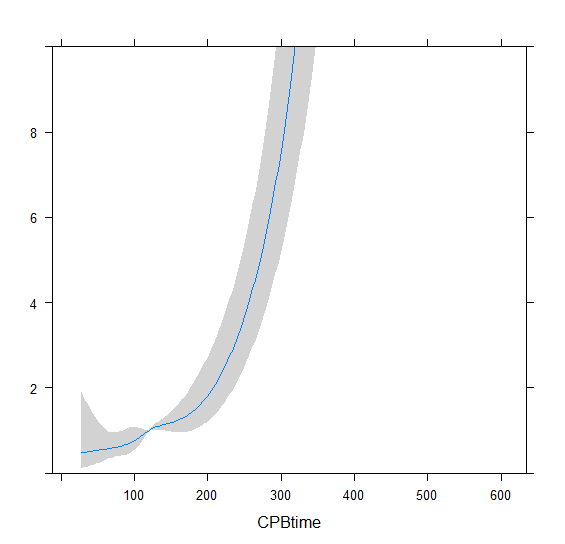

Supplement: Supplementary file 1 [file 2153-8174-24-2-038-s1.zip › 2153-8174-24-2-038-s1.docx]
